# Development of Healthy Protein-Rich Crackers Using *Tenebrio molitor* Flour

**DOI:** 10.3390/foods11050702

**Published:** 2022-02-26

**Authors:** Anna Djouadi, Joana Rides Sales, Maria Otília Carvalho, Anabela Raymundo

**Affiliations:** 1 National Institute of Agronomic Sciences for Food and the Environment, AgroSup Dijon, University of Bourgogne Franche-Comté, 21000 Dijon, France; anna.djouadi@agrosupdijon.fr; 2 LEAF—Linking Landscape Environment, Agriculture and Food, Instituto Superior de Agronomia, Universidade de Lisboa, Tapada de Ajuda, 1349-017 Lisboa, Portugal; joanasales@isa.ulisboa.pt (J.R.S.); motiliac@isa.ulisboa.pt (M.O.C.)

**Keywords:** crackers, *Tenebrio molitor*, insects, physical properties, antioxidants, phenolics, proteins, sensory analysis

## Abstract

Entomophagy is still a widespread practice in Africa and Asia, although it is declining due to the westernization of diets. Today, the issue of its rehabilitation is underway; indeed, the nutritional economic and ecological stakes of this consumption are strategic. It can be considered an important way to face the scarcity of natural resources, environmental pressures due to the increasing world population, and demand for protein. *Tenebrio molitor* larvae flour was recently approved by the European Food Safety Authority (EFSA) as a novel food. The aim of the present work was to create protein-rich healthy cracker from insect flour, achieving the claim “source of protein” with a target market focused on the healthy products for consumption on the go. Contents of *T. molitor* flour from 2 to 20% (%*w*/*w*) were tested, using a previously optimized formulation and the comparison in terms of nutritional, physical, and sensory properties with a standard formulation was performed. *T. molitor* incorporation allowed an improvement in the nutritional profile of snacks, through an increase of 15% in protein content and an enrichment in minerals (namely potassium, phosphorus, copper, and zinc). The crackers containing a 6% of insect flour were the most appreciated by the panelists. The incorporation of *T. molitor* induced a reduction in firmness and an increase in crispness, resulting from the impact of the protein on the structure. This aspect has a positive impact with respect of the acceptance of snacks—70% of the panelists consider the possibility to buy the crackers with 6% enrichment. A darkening of the samples with the increase in the incorporation of *T. molitor* flour was also observed, accompanied by a reduction of about 20% of the L* values. Globally, insect protein can play an important role in redesigning food diets, making them more sustainable, with less environmental impact and equally balanced.

## 1. Introduction

Entomophagy, which refers to the consumption of insects by humans, is still a widespread practice in Africa and Asia, although it is declining due to the westernization of diets [1]. In Westernized countries, it has been gradually abandoned in favor of livestock farming, mainly for profitability reason [2]. Today, the issue of its rehabilitation is underway; indeed, the nutritional, economic, and ecological stakes of this consumption are strategic. The Food and Agriculture Organization of the United Nations (FAO) considers insects as a sustainable source of protein, as an alternative to animal protein, that can be seen as an important pathway to address the scarcity of natural resources, increasing environmental pressures, population growth, and demand for protein [1].

The consumption of insects by humans as an alternative to more conventional animal proteins, can present significant nutritional and environmental benefits. Insects contain an equivalent level of protein, and a high level of nutrients and unsaturated fats. In addition, insect production has less environmental impact and requires less land and water compared to other animal protein sources [1]. On the other hand, enriching the human diet through the inclusion of edible insects contributes to its nutritional improvement, and can directly contribute to the first three United Nations Sustainable Development Goals (poverty, hunger eradication, and quality of health—DSG 2, 3, and 12), and can also contribute to mitigate the effects of climate change. Indeed, as previously mentioned, its production is more sustainable compared to the production of other more traditional sources of animal protein in Western society [3]. It has already been found that enriching bread with insect meal contributes to the improvement of the nutritional quality, in terms of protein, of the final product [4,5,6]. However, the acceptance of insect-based ingredients and foods is a barrier to their consumption in Western societies, where insect consumption is not embedded in dietary habits. However, consumers would be willing to consume them in a less visible form, in modified products, indistinguishable in familiar and staple food products [7]. As a result, some insect species could be incorporated into food products in powder form, providing additional nutrients.

In this study, larvae of *Tenebrio molitor* (Coleoptera, Tenebrionidae), more commonly known as “mealworms”, were used. *T. molitor* larvae have been recently approved by EFSA [8] as a novel food ingredient, as dried whole insect or in powder form, in application of Regulation (EU) 2015/2283 [9] on novel foods. With this state, EFSA considers that: (i) “insects are regularly consumed in many parts of the world” and (ii) yellow mealworm is safe for health, within the proposed uses and use levels for human consumption. In addition, it is important to note that eleven other applications of insects are currently undergoing safety assessment by EFSA [9]. This reveals the strong impact that insect consumption will have in the near future.

As many studies have shown, mealworms are one type of potential ingredient that can be used to improve the nutritional value of foods, especially cereal-based products, such as bread [5], pasta [10], and extruded snacks [11]. As the snacking market is continuously growing and the consumer demand is increasingly oriented towards health-friendly products, it was decided to focus our study on this type of product [12]. Indeed, in recent years, a trend towards healthier foods has been observed, composed of more health-friendly ingredients, in the lifestyle of consumers [13].

The aim of this study was to evaluate the impact of insect flour incorporation on the appearance, physical properties (texture), biochemical composition, as well as the antioxidant and sensory properties of wheat-based crackers, with the aim of obtaining a final product with the claim “protein source” or “high protein”. Crackers with different contents of Tenebrio molitor flour, ranging from 2% to 20% (%, *w*/*w*), were studied to obtain higher than usual contents of bioactive compounds. Indeed, according to several studies, mealworms are a source of protein. A study conducted by Zielińska et al. [14] found the following composition of mealworm larvae (per 100 g of dry sample): protein—52 g, fat—24 g, and mineral content—1g. However, the nutritional composition can vary depending on production conditions and life cycle. However, the nutritional composition can vary depending on production conditions and life cycle. Thus, different authors report results with some differences. Wemans [15] showed that mealworm larvae had ~45% crude protein, ~18% fat, and ~5% ash (dry matter). Another study conducted by Zhao et al. [16] revealed that *T. molitor* larvae contained about 51% crude protein, 33% fat, and 5% ash on a dry weight basis. González et al. [5] showed the nutritional composition of this species using wheat flour as food as ~49% of crude protein, 31% of fat, and ~4% of dry matter.

The protein fraction of insects has an adequate amino acid composition. This is indeed the case for *T. molitor* larvae, which have a particularly favorable composition, with relatively high amounts of essential amino acids, such as lysine and methionine, absent in cereal proteins. Regarding lipids, several studies have shown that *T. molitor* contains between 30 and 40% lipids, depending on its growth stage, with a larva being richer in lipids than an adult [5,17]. Another study conducted by FAO [1] showed that the composition of omega-3 and omega-6 unsaturated fatty acids in yellow mealworm larvae is comparable to that of fish (and higher than that of cattle and pigs), and the content of protein, vitamins, and minerals is like that of fish and meat.

On the other hand, the carbohydrate content is relatively low compared to wheat flour (around 70 g/100 g). Regarding the mineral content, like most insects, mealworms have been shown to be rich in potassium but low in calcium. However, it is possible to increase this amount of calcium by inserting a calcium-rich diet into the larva’s diet [18].

In the present work, it was intended to develop savory snacks (crackers) with incorporation of *T. molitor*, which can be used in the diet as an alternative protein source.

## 2. Materials and Methods

### 2.1. Production of T. molitor Flour

The snacks were produced from dried larvae of *T. molitor* (Linnaeus, Coleoptera: Tenebrionidae), gently supplied by Entrogreen (Santarém, Portugal), a Portuguese company pioneer in the production of insects for human consumption. *T. molitor* was grown on a nutrient medium composed of bran with flour or ground chicken feed, supplemented with carrots and apples, at a temperature between 18 °C and 20 °C, with a humidity of 70%. After egg laying by the adults, a wait period of 8 to 10 weeks was used before harvesting the larvae. The larvae underwent a specific diet to clean their intestine before harvesting. Indeed, the insects were not fed for 12 to 24 h after separation from the growth medium and before being killed. *T. molitor* larvae were separated from their nutrient medium by sieving and killed by boiling in hot water. Finally, the insects were rinsed and dried (microwave drying chamber, 1000 W, 18 min), to remove water and avoid potential microbial contamination. *T. molitor* larvae were ground into powder with a food processor (Bimby, Vorwerk), for 30 s at speed 7 (high speed level, scale from 1 to 10). Then, the flour was sieved to obtain a flour with a particle size with less than 100 μm.

### 2.2. Crackers Preparation

Crackers were prepared from a previously developed model formulation developed by Batista et al. [19], using wheat flour, water, a mixture of sunflower and corn oil, salt, and insect meal, as shown in Table 1. A control cracker, without insect flour, was also designed and analyzed. This model formulation has a reduced number of ingredients and a simple preparation, so that it can be easily and cheaply reproduced.

Batch sizes of 100 g were made, corresponding to approximately 30 crackers. All the ingredients were mixed by hand, using an optimized procedure, and then rolled out with a manual dough machine (generally used to produce pasta), reproducing the extrusion process (Atlas 150, Marcato, Italy) to a thickness of 1.8 mm. The crackers were then molded into jagged 38 mm squares and baked at 180 °C for 10 min in a convection oven Johnson A60 (Johnson & Johnson, New Brunswick, NJ, USA). To improve the crispness of the finished product, the crackers were dried in a stove 60 °C for 30 min (Arianna XLT133 (Unox, Cadoneghe, Italy). After cooling, some crackers (N = 10, 1/3 of the lot) were powdered and frozen for nutritional composition and other chemical analysis.

### 2.3. Crackers Dimensions

A digital caliper (model Z22855F, Powerfix, Pulloxhhill, Beldfordshire, UK) was used to evaluate the dimensions of the crackers; the width (W) and thickness (T) of 10 crackers of each formulation type were measured and the spread ratio (W/T) was calculated accordingly. The mass of the samples was also measured and the corresponding densities calculated (weight (g)/volume (cm^3^)). All these measurements were performed 24 h after the crackers preparation.

### 2.4. Color Analysis

The color of the crackers was instrumentally measured using a colorimeter (Minolta CR-400) with a D65 colorimetric standard and a visual angle of 2°. The results were expressed with the values of: L*, lightness including positive values from 0 to 100; a*, greenness (60 to −60 positive to negative); and b*, yellowness (60 to −60 positive to negative), according to the CIELab standard. The saturation, C*_ab_, was also calculated as follows: C*_ab_ = [(a*^2^ + b*^2^)]1/2.

All color measurements were also performed 24 h after baking, under the same lighting conditions and with a standard white with the following parameters: L* = 94.61, a* = 0.53 and b* = 3.62. The measurements were repeated 10 times for each formulation (one measurement per cracker).

### 2.5. Texture Analysis

The texture of the crackers was characterized, 24 h after baking, with a TA.XTplus texturometer (Stable Micro Systems, Godalming, UK), by a penetration test (5 mm distance), on a perforated base, using a stainless probe of 2 mm diameter with a speed of 3 mm·s^−1^, with a 5 kg load cell at room temperature (20 ± 2 °C). Hardness was calculated as the peak force (N) in the force versus time texturogram. This peak corresponds to the maximum force required to break the cracker. Crispiness was also determined from the same graph, and it is considered as the time needed to reach the maximum peak(s). The shorter the time in which the break occurs, the crispier the material. So, crispiness can be obtained from the time needed to break the cracker, which is inversely related to crispiness; the faster the breakage occurs, the crispier the cracker will be [20]. Measurements were repeated ten times for each sample of the different formulations (one measurement per cracker) as well as for the control.

### 2.6. Sensory Analysis

Crackers were evaluated by un untrained sensory panel (*n* = 56, age = 12–62, male = 18, female = 38) to assess which concentration of insect flour was most appreciated. To do so, the crackers containing 6% and 15% *T. molitor* flour were presented randomly, together with a control cracker. All tasters were previously informed that the crackers contained edible insects, approved by the EFSA. The cracker samples were evaluated in terms of color, smell, taste, texture, and overall liking (using six levels hedonic scale, ranging from “very pleasant” to “very unpleasant”). Purchase intention was also assessed, with five levels ranging from “I would definitely buy” to “I would definitely not buy”. The tests were carried out in a standardized sensory analysis room, according to EN ISO 8589 [21].

### 2.7. Determination of Water Content and Water Activity (a_w_)

The water content and water activity of the crackers were determined. Water content was measured by the loss of weight of a sample when heated to 100–105 °C, until a constant weight was obtained. Water activity (a_w_) was determined using a thermo-hygrometer (HygroPalm HP23-AW, Rotonic AG) at 20 ± 1 °C. The measurements were performed in triplicates from powdered samples.

### 2.8. Biochemical and Mineral Composition

The nutritional composition of *T. molitor* flour and crackers were performed accordingly with AOAC procedures [22]. The protein content was determined using a DUMAS protein/nitrogen analyzer (VELP Scientific NDA 702 DUMAS Nitrogen Analyzer—TCD detector), according to the Dumas method. The total nitrogen content was determined, and that value was multiplied by a conversion factor of 6.25 to obtain the crude protein content of the crackers [23,24]. The fat content was quantified according to the procedure used for cereals and derived products described by the Portuguese standard method NP4168. This method is based on the hydrolysis of the bonds between lipids, proteins, and carbohydrates, using hydrochloric acid, ethanol, and formic acid. The amount of lipids was determined gravimetrically, after evaporation of the solvent by oven drying. The total ash was measured gravimetrically by incineration at 550 °C in a muffle furnace, for 24 h. For the quantitative determination of all elements (Cu, Na, K, Fe, Ca, Zn, Mn, Mg, and P), an acid digestion of the sample (about 0.5 g) was performed using a mixture of HNO_3_ and HCl (3:1) at 105 °C (with staged heating) in a DigiPrep MS digester (SCP Science, Baie-d’Urfé, QC, Canada). The determination of mineral elements was performed by atomic absorption spectrophotometry and ICP-OES (iCAP 7000 series, Thermo Scientific, Waltham, MA, USA) [25]. All analysis were repeated 3 times. Carbohydrates were determined by the calculation: (100 − [protein + fat + ash + water content]). The total energy value was calculated by adding the proteins, lipids, and carbohydrates, using their conversion factors as given in Annex XIV of Regulation (EU) No. 1169/2011 [26].

### 2.9. Total Phenolic Compounds and Antioxidant Capacity Determination

For total phenolic content and antioxidant capacity quantification, extracts were prepared according to the procedure used by Barreira et al. [27] and Reis et al. [28].

Polyphenols were quantified using the Folin−Ciocalteu reagent according with the procedure described by Mohankumar et al. [29]. This method is based on a redox reaction, and the Folin−Ciocalteu reagent is composed of polyheterocycles which is a mixture of phosphotungic acid and phosphomolibdic acid. This reagent is reduced during the oxidation of polyphenols, which produces a blue coloration due to the formation of a complex of molybdenum and tungsten, whose absorbance is proportional to the quantity of polyphenols contained in the extract. Then, 150 µL of the sample from the extraction was added to 2.4 mL of distilled water and 140 µL of Folin−Ciocalteu reagent. After 3 min of reaction, 300 µL of 1 M sodium carbonate was added. The solution was incubated in the dark for 2 h at room temperature. The absorbance was measured at 725 nm. The results were calculated using the standard curve of gallic acid (0 to 200 µg·mL^−1^) and are expressed in mg of equivalent gallic acid per gram of sample (mg EAG·g^−1^).

To evaluate the antioxidant activity, two methods were used. The DPPH method was performed according to the method described by Brand-Williams et al. [30], with some modifications. This test is based on the reduction of the stable DPPH° radical dissolved in methanol to DPPH-H by the antioxidants included in the studied extract. A quantity of 100 µL of extract was added to 3.9 mL of DPPH solution. The mixture was incubated in the dark at room temperature for 40 min. The absorbance was measured at 515 nm and the results were calculated from a standard curve of Trolox (6-hydroxy-2,5,7,8-tetramethylchroman-2-carboxylic acid) (0 to 1000 µmol·L^−1^).

FRAP method described by Benzie and Strain [31] was also used. This method is based on the reduction of ferric iron Fe^3+^ from potassium ferricyanide in the TPTZ (2,4,6-tri(2-piridil)-1,3,5-triazine) reagent to ferrous iron Fe^2+^ in the presence of antioxidant, whereby 90 µL of the sample was mixed with 270 µL of distilled water and 2.7 mL of FRAP reagent. After mixing, the solution was placed in a water bath at 37 °C for 30 min. The absorbance was measured at 595 nm and the results calculated from a Trolox standard range (0 to 700 µmol·L^−1^).

For both methods, the results are expressed as mg of Trolox per mg of dry extract. All analysis were repeated in triplicate and performed on powdered cracker samples and Tenebrio flour.

### 2.10. Microbiological Analysis

To control the quality of the finished product, an enumeration on GYP medium (glucose, yeast extracts, peptone) was carried out for the cracker with 6% *T. molitor* flour and on the pure flour. Results were obtained from a range of dilutions in liquid medium, from 10^−1^ to 10^−3^ [32]. Enumeration for each sample and dilution was duplicated.

### 2.11. Statistical Analysis

All results were statistically analyzed using Prism 5 software and subjected to analysis of variance (one-way ANOVA) and Tukey’s test with a margin of error of 5% (*p* < 0.05). All results were presented as mean ± standard deviation.

## 3. Results

### 3.1. Color Analysis

**Figure 1:**
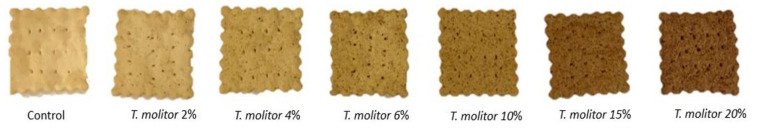
Appearance of crackers containing 2 to 20% *T. molitor* flour (% *w*/*w*) and the control.

**Figure 2:**
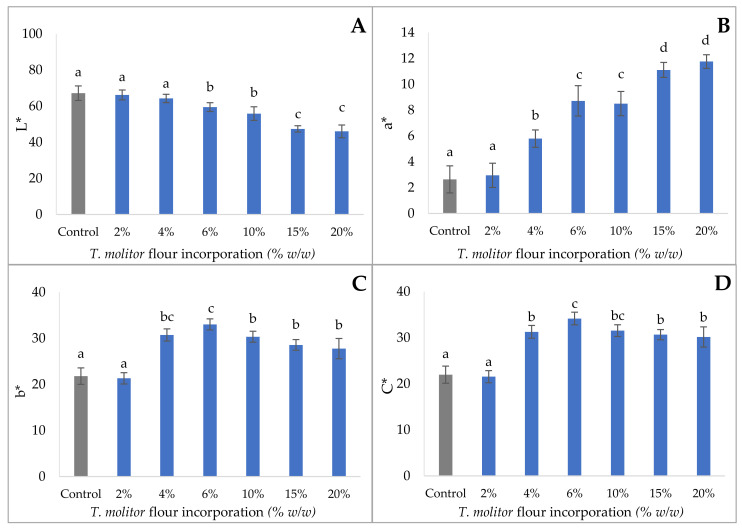
Brightness (L*) (**A**), greenness (a*) (**B**), yellowness (b*) (**C**), and chroma (C*) (**D**) of crackers containing 2–20% *T. molitor (w/w)*. Results are expressed as mean ± standard deviation (*n* = 10). Different letters indicate significant differences (*p* < 0.05) between different concentrations of *T. molitor*.

The appearance of the crackers is shown in Figure 1. It was observed that the greater the amount of insect flour, the darker the cookie appears. This was expected since insect flour is darker than the wheat flour, usually used for this type of crackers. The evolution of the color parameters, in terms of brightness (L*), greenness (a*), yellowness (b*), and saturation (C*), is shown in Figure 2.

Regarding the brightness (L*) of the product, a decrease was observed inversely to the amount of insect flour incorporated. Indeed, the L* value of the control cracker (67.18) is significantly higher (*p* < 0.05) from those containing 4%, 6%, 10%, 15%, and 20% of *T. molitor* flour; a reduction of about 20% of L* value was observed for 15 and 20% of Tenebrio flour incorporation. On the contrary, the incorporation of *Tenebrio* flour induces an increase in the color intensity level from red to green (a*). The control cracker a* value (2.64) is significantly lower than the crackers with incorporations of 4%, 6%, 10%, 15%, and 20%. Numerous studies have shown similar results for the enrichment of bread [5], muffins [33], cookies [34], and shortcake [35] with *T. molitor* flour. These results can be explained on the one hand by the formation of Maillard reaction compounds, favored by the presence of insect flour with high protein content (Figure 1) and, on the other hand, due to the water loss and volume change induced by baking that strongly induce the color of crackers. Regarding the saturation of the crackers, the enriched ones have more saturated colors than the control (higher C* values, changing from 20 for the control to 30 for the highest level of incorporation). This means that the color went from reddish to yellowish.

For all parameters, the incorporation of a small amount of insect flour (2%) does not significantly (*p* > 0.05) influence the color of the finished product.

### 3.2. Crackers Dimensions

The characteristic dimensions of the crackers are shown in Table 2. In general, no significant difference was observed in the width of the crackers. For the thickness, only the crackers with 2% and 4% are not significantly different from the control. Above these concentrations, a significant decrease in thickness was observed (*p* < 0.05) and a similar effect was observed for spread parameter. It was noticed that the spread is higher when there was more insect flour is incorporated. However, regarding the density of the crackers, no significant difference was observed up to 15% incorporation. This is a relevant quality parameter, as consumers generally want less heaviness and more density [36]. This suggests that the presence of *T. molitor* does not alter the gas retention in the crackers, which is an important phenomenon related to the cracker’s texture and sensory attributes.

### 3.3. Texture Analysis

**Figure 3:**
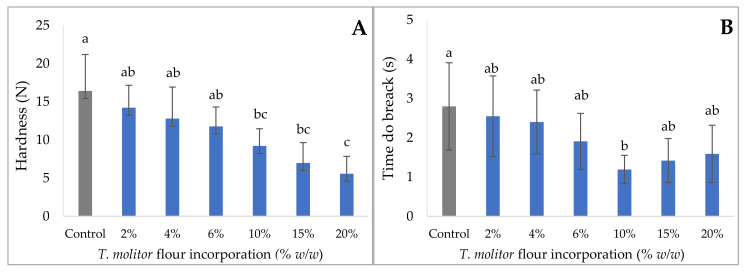
Hardness (N) (**A**) and time to break (s) (**B**) of crackers with 2% to 20% *T. molitor* flour incorporation. Results are expressed as mean ± standard deviation (*n* = 10). Different letters indicate significant differences (*p* < 0.05) between different concentrations of *T. molitor*.

When developing cracker-type snacks, one of the most important parameters is the texture of the final product. When consuming this type of product, texture that crunches under the tooth are generally appreciated [37]. In order to evaluate the impact of insect flour incorporation on texture properties, a penetration test was performed. In this test, the cracker was placed on a tray that included a hole, and then the probe exerts a normal tension that leads to breakage in the center. The results of the hardness and time required for break are shown in Figure 3.

In general, a decrease in hardness was observed with the incorporation of insect flour—a reduction around 30% in the firmness values was observed, comparing the control and the crackers with 15% incorporation. In fact, the more *T. molitor* content flour present, the less force required to cause the breakage, i.e., meaning that the crackers become softer. This decrease in hardness is accompanied by a reduction in the breakage time of the structure, i.e., the breakage of the structure occurs earlier, which is associated with greater crispiness. Indeed, the addition of insect flour disturbs the formation of the gluten network responsible for the homogeneity of the dough. This was prominent during the realization of the dough, which was more difficult to mix and to handle. Similar results were observed, especially in the study of Zielińska et al. [38] on muffin enrichment with *T. molitor* and cricket flour. This study showed that mealworm enrichment resulted in much softer crackers, but also in elasticity, resilience, cohesion, and chewiness of muffins, except for 6% and 10% flour incorporation (as in our study). However, this study shows that mealworm flour has a more significant impact on the overall texture of the product.

### 3.4. Sensory Analysis

**Figure 4:**
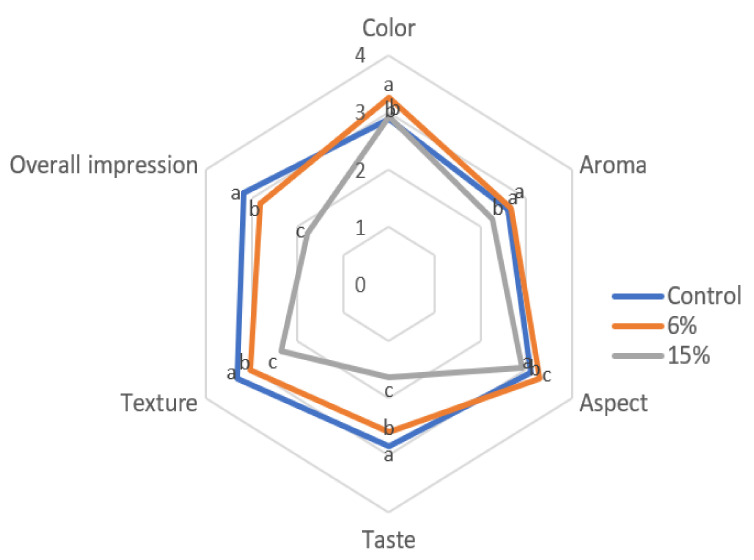
Panelist responses from the sensory evaluation (*n* = 56) of crackers enriched with 6% and 15% *T. molitor* flour as well as the control sample. The sensory attributes were classified as follows: 0—very unpleasant; 1—unpleasant; 2—indifferent; 3—pleasant; and 4—very pleasant. Different letters indicate significant differences (*p* < 0.05) between different concentrations of *T. molitor*.

According to the type of sensory analysis performed (untrained panel), more than three samples lead to results that are difficult to compare. Thus, according to the texture results, it is clear that incorporations of more than 6% result in much softer crackers. On the other hand, the crackers with 20% of Tenebrio flour were too dark and had a bitter taste; thus, its sensory appreciation was not considered acceptable. So, sensory evaluation on the snack crackers containing 6% and 15% *T. molitor* flour and the control was conducted. Figure 4 represents the average scores of the sensory parameters considered.

**Figure 5:**
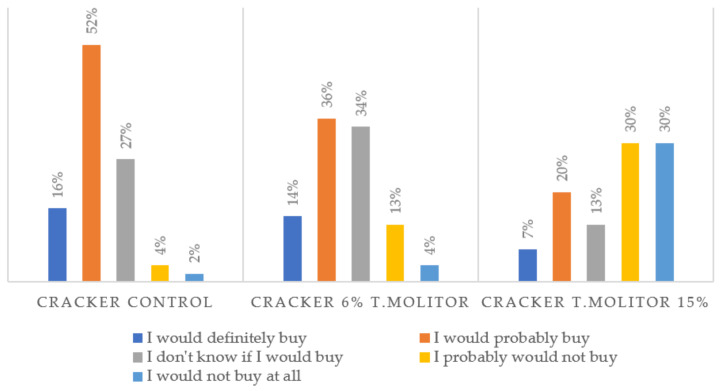
Sensory evaluation panelists’ responses (*n* = 56) in terms of purchase intention for crackers enriched with 6% and 15% (% *w*/*w*) *T. molitor* flour, as well as the control sample.

Generally, the control sample was preferred to those containing insect flour. It should be noted that even the panelists did not assign high scores to the standard. Indeed, it obtained the highest score for most sensory attributes except for color, appearance, and aroma. For these three attributes, the cracker with 6% of insect flour incorporation obtained the highest score (3.27, 2.66, and 3.30, respectively, for color, appearance, and aroma, where 3 = pleasant). Moreover, 70% of the panelists assumed that they would be willing to buy the crackers with 6% *Tenebrio* flour (Figure 5). However, purchase intentions decrease drastically with increasing concentration of insect meal; 60% of the tasters would probably not be available to buy the crackers with 15% incorporation. Similar results were observed in the study by Zielińska et al. [38], on the development of muffins enriched with *T. molitor* flour. The cracker containing a significant amount of insect flour was poorly appreciated by the consumer, especially in terms of taste and texture.

Considering the results obtained for the sensory analysis, all the experiments that followed were performed on the cracker most appreciated by the panel, i.e., the cracker enriched with 6% *T. molitor* flour, in comparison with the control.

### 3.5. Total Water Content and Water Activity (aw) Determination

Table 3 summarizes the water content and water activity of the crackers enriched with *T. molitor* flour. These values are quality parameters. Indeed, for this type of food, the water content and water activity strongly influence the crispness of the product, its sensory acceptance, and its shelf life. A study conducted by Arimi et al. [39] shows that, beyond a critical value of water activity (around 0.5), foods become softer and stale, thus losing their crispness.

A significant decrease in water content was observed in the cracker enriched with insect flour, compared to the control (from 0.162 to 0.132). The water present in the dough comes only from the water added to the system (according to the formulations presented) and from the water content of the raw materials. The proportional change in water content was, therefore, caused by the replacement of wheat flour with *T. molitor* flour, which has a lower water content than the other ingredients. As mentioned before, it is possible that the addition of high concentrations of insect flour leads to a weaker gluten network, unable to effectively trap gas bubbles and water molecules, resulting in a decrease in product moisture. Similar results were observed in studies conducted by Min et al. [34] and Zielińska et al. [35] on the enrichment of shortcake with *T. molitor* flour. Likewise, there is a consistent reduction in water activity with the addition of *Tenebrio* flour. Likewise, there is a consistent reduction in water activity with the addition of *Tenebrio* flour. Crackers showed aw values around 1.6, meaning that, in general, the addition of insect flour imparts a positive impact on the shelf life of the crackers (and the consequent loss of crispness). These results may suggest that wheat flour has a greater water holding capacity than *T. molitor* flour. The aw values obtained are low (<0.17), which is associated with a longer shelf life, since spoiling bacteria do not have optimal conditions to grow [40].

### 3.6. Biochemical and Mineral Composition Determination

The biochemical and mineral composition of the crackers was reported in Table 4. As edible insects are known to be protein rich foods, the raw *T. molitor* meal was determined to be 51% protein of dry weight, which is relatively close to the values found in the literature [14,18,41]. The protein content in the cracker enriched with 6% insect meal increased by 4.3% compared to the control. This effect was expected, as worm flour is the richest in protein among all ingredients in the recipe, so increasing its content leads to a proportional increase in protein content in the final product. According to the European regulation (EU) 1924/2006 [42], the finished product, containing 6% of *T. molitor* flour, can use the claim “source of protein”, since its protein content represents more than 12% of the total energy. However, the amount of protein does not allow the mention “rich in protein”. There was also a significant increase (*p* < 0.05) in the amount of total ash (2.18%), which reflects the increase in mineral content.

In terms of fat content, no significant differences (*p* > 0.05) were observed between the control and enriched crackers, with crude fat contents ranging from 11.1% to 12.7% (Table 4).

In general, there was a progressive increase in protein (from 1.90 to 2.18 g/100 g) and ash (from 1.90 to 2.18 g/100 g) content, and a decrease in carbohydrate content (from 72.7 to 71.0 g/100 g) as the concentration of worm flour increased, which is associated with a reduction in the calorie intake.

Regarding the mineral profile, the results are listed in Table 5. It was observed that pure *T. molitor* flour is a source of potassium, magnesium, phosphorus, and iron. This induces similar results in crackers containing it. According to the recommended daily values (RDI) established by the European Regulation N°1924/2006; Directive N 90/494 (EC) [42], the cracker enriched with 6% of *T. molitor* flour can be a “source of potassium, magnesium, phosphorus, and iron”. In terms of sodium, a non-significant (*p* > 0.05) increase was observed. In comparison with literature, similar results were observed for pure *Tenebrio molitor* flour. In fact, the study carried out by Costa et al. [43] about the characterization of *Tenebrio* flour presents results with the same order of magnitude, especially in potassium (800 mg/100 g), magnesium (282.3 g/100 g), phosphorus (797.0 mg/100 g), copper (0.78 mg/100 g), zinc (9.65 mg/100 g), and manganese (1.10 mg/100 g). Similar values are stated in the supported document published by EFSA [8], on the approval of *T. molitor* as a novel food [9].

Considering the values of 15% of RDV summarized in the table, of each one of the minerals, it is possible to verify that the crackers enriched with *Tenebrio* may present a nutritional claim “source of” in the case of potassium, phosphorus, iron, copper, zinc, and manganese, since they have contents higher than 15% of DVR (highlighted in the table), according with the Regulation (CE) No 1924/2006 [42].

### 3.7. Total Phenolic Compounds and Antioxidant Capacity

**Figure 6:**
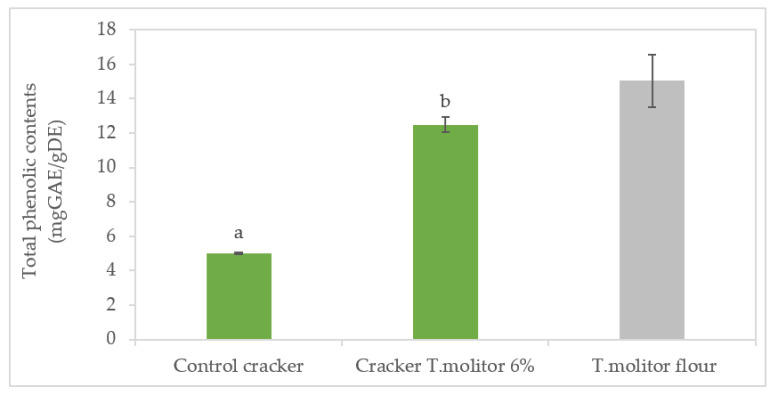
Total phenolic compound content (expressed as gallic acid equivalents mg·g^−1^ dry extract) of crackers enriched with different levels of insect flour incorporation (green) and *Tenebrio* flour (gray). Results are expressed as mean ± standard deviation (*n* = 3). Different letters correspond to significant differences (*p* < 0.05) between samples.

These phenolic compounds make a decisive contribution to the antioxidant capacity; from Figure 6, it is clear that the incorporation of insect flour caused a very significant increase (*p* > 0.05) in the total phenolic compounds (more than double compared to the control—from 5.02 mg GAE·g^−1^ to 12.49 mg GAE.g^−1^). However, since Tenebrio flour has a content of total phenolic compounds of 15 mg GAE·g^−1^, it can be expected that the value of phenolic compounds may be overestimated in crackers with 6% of Tenebrio flour. This fact may result from the interference of the protein in the quantification of phenolic compounds, by the Folin−Ciocalteu reagent. It should also be noted that the control sample (9.65 g/100 g protein) already has a considerable content of phenolic compounds (5.02 mg GAE·g^−1^).

It is important to evaluate the antioxidant capacity of our final product, since the presence of antioxidants plays an important role in our diet. These compounds play a role in the prevention of diseases related to oxidative stress, in cardiovascular diseases, diabetes, or the aging process, which are all among the major public health problems nowadays [44]. In addition, if our product contains antioxidants in sufficient quantity, it ensures the stability of the product over time because, as its name suggests, these molecules will protect the snack cakes from oxidation.

**Figure 7:**
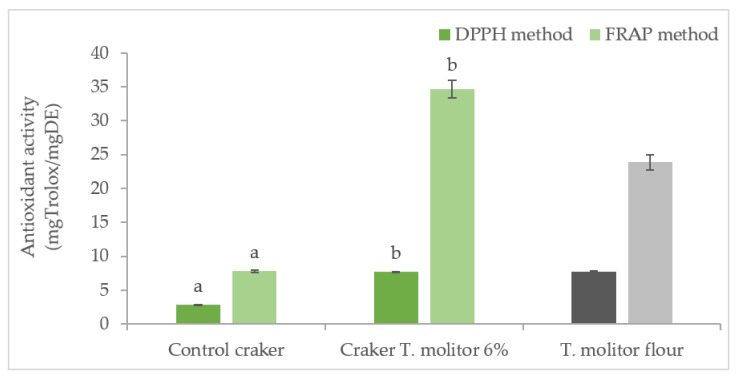
Antioxidant capacity of crackers (expressed as mg Trolox per mg dry extract of fortified crackers) with different levels of insect flour (green) and *T. molitor* flour. Dark bars for the DPPH method and light bars for the FRAP method. Results are expressed as mean ± standard deviation (*n* = 3). Different letters correspond to significant differences (*p* < 0.05) between samples.

The antioxidant properties of the crackers and flour were evaluated in accordance with the ability to neutralize the DPPH radical and the measurement of reducing power (FRAP). The overall results are presented in Figure 7.

Regarding the antioxidant activity accessed by DPPH radical capture, a significant increase (*p* < 0.05) was observed when the insect flour was incorporated. Similar results were observed by ferric ion reduction (FRAP method).

Navarro del Hierro et al. [45] studied the DPPH scavenging activity of mealworm extracts and also confirmed their strong antioxidant properties. As expected, partial substitution of wheat flour with worm flour significantly (*p* < 0.05) increased the free radical scavenging capacity, as indicated by the DPPH scavenging activity. In addition, numerous studies have identified bioactive compounds in insects, such as polyphenols, chitins, peptides, and proteins. Since insects are known to be high in protein and are potential sources of bioactive proteins and peptides responsible for antioxidant activity [44,46,47].

Depending on the type of edible insects, these proteins may change the DPPH radical scavenging activity and the reducing power of ferric ion. These changes may depend on the molecular weight of the protein or peptide in question, as well as the amino acid composition [48]. Considering the high radical scavenging activity of DPPH, the results obtained suggest that mealworm proteins contain amino acids or peptides that act as electron donors and can react with free radicals to transform them into more stable compounds. It is also important to note that the increase in antioxidant activity observed with the FRAP method is greater for the 6% cracker than for pure *Tenebrio* flour. This fact can also be explained by the existence of the Maillard reactions already mentioned, which also originated compounds with high antioxidant power. These reactions occur during food processing at high temperatures (as baking), between amine groups (from proteins) and carbonyl compounds (from reducing sugars), inducing the formation of compounds with antioxidant activity, such as reactive oxygen species scavenging activity or metal-chelating activity. Although some of the compounds formed in Maillard reactions have a negative impact on health, when consumed regularly and in large amounts, their contribution to an increase in antioxidant capacity has been duly studied by several authors, such as Shen et al. [49] who studied the Maillard reaction manipulation to maximize the antioxidant potential of white bread products.

### 3.8. Determination of Microbiological Activity of 6% Crackers and Raw Flour

The counts of molds and yeasts in the crackers with 6% *T. molitor* incorporation and in the raw flour were evaluated. The total mold and yeast contents are relatively close for the 6% cracker and the raw flour (4.26 and 4.91 log cfu·mL^−1^ respectively). Similar results were found for *T. molitor* flour by Vandeweyer et al. [50] (4.5 log cfu·mL^−1^) and in the study by Costa et al. [43] on the evaluation of *T. molitor* as a new food source (4.4 log cfu·mL^−1^). In order to have more in-depth results on the microbiological aspect of *T. molitor*, it would have been judicious to also count bacterial colonies (total viable aerobic and anaerobic), as well as to search for certain microorganism-types responsible for food infection (*Salmonella* spp., *Escherichia coli*, *Staphylococcus*, *Listeria monocytogenes*). Indeed, several studies have demonstrated the presence of Enterobacteriaceae, i.e., 6.3 log cfu·mL^−1^ and 7.6 log cfu·mL^−1^ by Costa et al. [43] and Wynants et al. [51], respectively, in *T. molitor* larvae. These values are higher than the legal limit set for minced meat or raw material used in the preparation of meat [52]. This limit (3 log cfu·mL^−1^ for enterobacteria) is, according to some authors, applicable to insects because there is no specific regulation for their consumption. However, only the Belgian Higher Health Council (HSC) (Brussels, Belgium) and the Federal Agency for the Safety of the Food Chain (FASFC) (Brussels, Belgium), as well as the Dutch Food and Consumer Product Safety Authority (NVWA) (Utrecht, The Netherlands), have written an opinion on the food safety aspects of edible insects [53]. These opinions are based on microbial counting and refer to food safety regarding *Salmonella, Listeria monocytogenes*, and *Escherichia coli* in meat and shellfish dishes. Thus, the detection of *Salmonella* spp. and *L*. *monocytogenes* must lead to an undetectable amount in 25 g of sample. These results were notably found by Vandeweyer et al. [50] and Costa et al. [43] in their respective studies.

## 4. Conclusions

The present work highlighted the interest of incorporating *T. molitor* flour in cracker-type snacks in order to enhance their nutritional profile. A maximum level of 6% (*w*/*w*) incorporation was accepted in terms of sensory evaluation. However, for this incorporation level, the nutritional qualities of the cracker, especially in terms of proteins and minerals, was achieved, with a slight impact on textural properties of the product. As *T. molitor* flour is rich in proteins and certain minerals, its incorporation in the appetizer crackers has allowed the claim “source of protein” and “source of potassium, magnesium, phosphorus iron and zinc”. The use of this flour is also interesting in terms of the antioxidant capacity of the product.

The enrichment of *T. molitor* flour also affected the color of the crackers (20% decrease of the brightness L* values); however, for low incorporation levels (less than or equal to 6%), the resulting browning was well accepted, without altering the overall appreciation by the panelists. In fact, the crackers with 6% (*w*/*w*) of insect flour were the most appreciated by the panelists, as a large part of them (70%) even said that they were ready to buy them. These results are relatively satisfactory and suggest that insect-based crackers could become widely appreciated and consumed as functional foods in the near future in Western countries, where insect consumption is still low.

The obtained results support the idea of using *T. molitor* flour as an ingredient with the potential to promote the nutritional profile of a food. This sustainable alternative source of food can be considered a positive impact in nutritional terms and can represent an alternative to the protein shortage in future decades all over the world.

## Figures and Tables

**Table 1:** Cracker formulation (*w*/*w*). F1—formulation of control crackers; F2—formulation of crackers with 2% *T. molitor* flour; F3—formulation of crackers with 4% *T. molitor* flour; F4—formulation of crackers with 6% *T. molitor* flour; F5—formulation of crackers with 10% *T. molitor* flour; F6—formulation of crackers with 15% *T. molitor* flour; F7—formulation of crackers with 20% *T. molitor* flour.

Ingredients	F1	F2	F3	F4	F5	F6	F7
	g/100 g	g/100 g	g/100 g	g/100 g	g/100 g	g/100 g	g/100g
Wheat flour	62	60	58	56	52	47	42
Water	28.5	28.5	28.5	28.5	28.5	28.5	28.5
Oil	8.5	8.5	8.5	8.5	8.5	8.5	8.5
Salt	1.0	1.0	1.0	1.0	1.0	1.0	1.0
*T. molitor*	0.0	2.0	4.0	6.0	10.0	15.0	20.0

**Table 2:** Characteristic dimensions of crackers with *T. molitor* flour incorporation from 2% to 20% (% *w*/*w*). Results are expressed as mean ± standard deviation (*n* = 10). Different letters in the same column correspond to significant differences (*p* < 0.05) between different concentrations of *T. molitor*.

	Width (W) (mm)	Thickness (T) (mm)	Spread Ratio (W/T)	Density (g/cm^3^)
Control cracker	37.4 ± 0.52 ^a^	4.00 ± 0.76 ^a^	9.7 ± 1.8 ^a^	7.7 ± 1.7 ^a^
Cracker *T. molitor* 2%	37.5 ± 0.74 ^a^	3.467 ± 0.50 ^ab^	11,0 ± 1.5 ^ab^	8.2 ± 1.3 ^ab^
Cracker *T. molitor* 4%	38.0 ± 0.63 ^a^	3.34 ± 0.49 ^ac^	11.6 ± 1.9 ^ac^	8.8 ± 1.7 ^ab^
Cracker *T. molitor* 6%	37.3 ± 0.72 ^a^	3.03 ± 0.47 ^bcd^	12.6 ± 2.0 ^bcd^	9.1 ± 1.3 ^ab^
Cracker *T. molitor* 10%	37.8 ± 0.50 ^a^	2.75 ± 0.50 ^ce^	14.1 ± 2.3 ^cd^	9.1 ± 1.4 ^ab^
Cracker *T. molitor* 15%	37.1 ± 0.63 ^a^	2.47 ± 0.31 ^de^	15.2 ± 2.1 ^de^	9.2 ± 1.4 ^ab^
Cracker *T. molitor* 20%	37.2 ± 0.90 ^a^	2.17 ± 0.32 ^e^	17.5 ± 2.1 ^e^	9.7 ± 1.3 ^b^

**Table 3:** Water content (moisture) and water activity (a_w_) of crackers with 6% *T. molitor* flour incorporation in comparison with the control cracker and pure flour. Results are expressed as mean ± standard deviation (*n* = 3). Different letters in the same column correspond to significant differences (*p* < 0.05) between samples.

	Water Content (g/100 g)	Water Activity (a_w_)
Cracker control	3.0 ± 0.1 ^a^	0.162 ± 0.008 ^a^
Cracker *T. molitor* 6%	1.8 ± 0.17 ^b^	0.132 ± 0.01 ^b^
*T. molitor* flour	6.3 ± 0.6	0.527 ± 0.001

**Table 4:** Biochemical composition (g/100 g) of crackers with 6% and pure *T. molitor* meal incorporated (*m*/*m*). Results are expressed as mean ± standard deviation (*n* = 3). Different letters in the same column correspond to significant differences (*p* < 0.05) between samples.

	Ash (g/100 g)	Total Fat (g/100 g)	Protein (g/100 g)	Carbohydrate (g/100 g) *	Total Energy (Kcal/100 g)
Cracker control	1.90 ± 0.03 ^a^	12.7 ± 6.9 ^a^	9.65 ± 0.13 ^a^	72.7	443.9
Cracker *T. molitor* 6%	2.18 ± 0.03 ^b^	11.1 ± 0.5 ^a^	13.90 ± 0.65 ^b^	71.0	439.3
*T. molitor* flour	3.40 ± 0.01	20.0 ± 1.2	51.20 ± 1.76	19.3	462.0

* Carbohydrates were calculated by difference from the average ash, fat, protein, and waters contents.

**Table 5:** Mineral composition (mg/100 g) of crackers with 6% and pure *T. molitor* flour incorporated (% *w*/*w*). Results are expressed as mean ± standard deviation (*n* = 3). Different letters in the same line correspond to significant differences (*p* < 0.05) between samples. Recommended daily value (RDV) per European Community Regulation N,1924/2006, Directive N-9090/494 (CE) [42].

	15% RDV (mg/100 g)	Cracker Control (mg/100 g)	Cracker *T. molitor 6%* (mg/100 g)	*T. molitor* Flour (mg/100 g)
**Na**	300	639.93 ± 5.35 ^a^	656.80 ± 20.69 ^a^	131.47 ± 10.05
**K**	225	203.14 ± 2.10 ^a^	**278.17 ± 6.24 ^b^**	917.8 ± 10.71
**Ca**	120	20.89 ± 0.24 ^a^	22.60 ± 1.62 ^b^	55.63 ± 11.66
**Mg**	56.2	24.29 ± 0.02 ^a^	43.91 ± 2.07 ^b^	238.29 ± 4.41
**P**	105	96.23 ± 0.72 ^a^	**152.14 ± 1.81 ^b^**	740.09 ± 6.98
**Fe**	2.2	2.62 ± 1.69 ^a^	**3.80 ± 1.66 ^a^**	19.38 ± 21.75
***Cu***	0.2	0.24 ±0.04 ^a^	**0.35 ± 0.005 ^b^**	2.00 ± 0.07
***Zn***	1.5	0.83±0.02 ^a^	**2.01 ± 0.04 ^b^**	14.18 ± 0.23
***Mn***	0.4	0.78±0.01 ^a^	**0.67 ± 0.02 ^b^**	1.28 ± 0.18

## Data Availability

Data is contained within the article.
